# The Relationship among Gene Expression, the Evolution of Gene Dosage, and the Rate of Protein Evolution

**DOI:** 10.1371/journal.pgen.1000944

**Published:** 2010-05-13

**Authors:** Jean-François Gout, Daniel Kahn, Laurent Duret, Paramecium Post-Genomics Consortium

**Affiliations:** Laboratoire de Biométrie et Biologie Evolutive, Université de Lyon, Université Lyon 1, CNRS, INRA, INRIA, UMR 5558, Villeurbanne, France; University of Chicago, Howard Hughes Medical Institute, United States of America

## Abstract

The understanding of selective constraints affecting genes is a major issue in biology. It is well established that gene expression level is a major determinant of the rate of protein evolution, but the reasons for this relationship remain highly debated. Here we demonstrate that gene expression is also a major determinant of the evolution of gene dosage: the rate of gene losses after whole genome duplications in the Paramecium lineage is negatively correlated to the level of gene expression, and this relationship is not a byproduct of other factors known to affect the fate of gene duplicates. This indicates that changes in gene dosage are generally more deleterious for highly expressed genes. This rule also holds for other taxa: in yeast, we find a clear relationship between gene expression level and the fitness impact of reduction in gene dosage. To explain these observations, we propose a model based on the fact that the optimal expression level of a gene corresponds to a trade-off between the benefit and cost of its expression. This COSTEX model predicts that selective pressure against mutations changing gene expression level or affecting the encoded protein should on average be stronger in highly expressed genes and hence that both the frequency of gene loss and the rate of protein evolution should correlate negatively with gene expression. Thus, the COSTEX model provides a simple and common explanation for the general relationship observed between the level of gene expression and the different facets of gene evolution.

## Author Summary

The analysis of gene evolution is a powerful approach to recognize the genetic features that contribute to the fitness of organisms. It was shown previously that selective constraints on protein sequences increase with expression level. This observation was surprising because there is *a priori* no reason why lowly expressed genes should be less important than highly expressed genes for the proper function of an organism. Here we show that selective pressure on the evolution of gene dosage, which is another important aspect of gene evolution, is also directly dependent on gene expression level. To explain these observations, we propose a model based on the fact that gene expression is a costly process (notably protein synthesis), so that there is an optimal expression level for each gene corresponding to a trade-off between the benefit and the cost of its expression. This model predicts that selective pressure on gene expression level or on the encoded protein should on average be stronger in highly expressed genes, providing a simple and common explanation for the general relationship observed between gene expression and the different facets of gene evolution.

## Introduction

Mutations can affect the phenotype either by modifying the sequences of proteins or by changing their pattern of expression. Whereas the evolutionary constraints acting on protein-coding sequences are relatively well characterized, those driving the evolution of gene expression have been much less studied. Modifications in gene expression can result from mutations in regulatory elements or through changes in the number of gene copies in the genome (*i.e.* gene dosage) by gene duplications or gene losses. The phenotypic impact of changes in gene dosage is clearly illustrated by the deleterious effects caused by chromosome aneuploidy [1]. The necessity of an X-chromosome inactivation mechanism to compensate for dosage imbalance between males and females in mammals [2] is another example of the importance of having the correct dosage of genes. Within populations, polymorphism in copy number of genes (Copy Number Variations: CNVs) significantly contributes to variations in transcript abundance [3]. Moreover, some CNVs were shown to be driven by positive selection for increased expression of the corresponding genes [4]–[6], highlighting the fact that gene dosage modifications can be targeted by selection. However, the evolutionary constraints that apply on gene dosage remain poorly understood.

Whole-genome duplications (WGDs) represent interesting cases to study the evolutionary constraints on gene dosage. Immediately after a WGD event, all genes are present in two copies; these paralogs that result from WGD are termed ohnologs, in reference to the pioneering ideas of Susumu Ohno on the role of WGDs in genome evolution [7], [8]. However progressive changes in gene dosage do occur: most ohnologs are lost, while only a subset is retained over long evolutionary times [9], [10]. Different (non-exclusive) models have been proposed to explain the retention of gene duplicates after a genome duplication. First, some ohnologs are retained because one or both copies evolved toward a different function, either by gain of a new function (neo-functionalization [7], [11]) or through partition of ancestral functions [12], [13 for review]. The over-retention of some functional categories suggests that WGDs might have played a role in some important evolutionary transitions by providing opportunities for functional innovations [14], [15]. Second, some ohnologs appear to be retained because of constraints on relative gene dosage (the ‘dosage balance’ hypothesis). For example, the loss of ohnologs encoding subunits of protein complexes is counter-selected because it affects the stoichiometry of complexes [16]–[18].

In yeast, it has been noticed that genes that have been maintained in two copies after WGD tend to be highly expressed [19]. However, the interpretation of this observation remained unclear: does it simply reflect an indirect effect of other parameters (*e.g.* differences in functional categories between highly and weakly expressed genes) or is there a direct relationship between expression and the probability of retention of ohnologs? The genome of *Paramecium tetraurelia*, which contains almost 40,000 protein-coding genes, provides a perfect configuration to investigate this issue. Indeed, 3 WGDs occurred during the evolution of the *Paramecium* lineage [17]. The genome contains about 12,000 pairs of ohnologs resulting from the most recent WGD, compared to less than 600 in yeast [20]. This corresponds to a frequency of gene loss of 49% since the last WGD (frequencies of gene loss after the intermediary and the old WGD are respectively 76% and 92%) [17]. Thus, the *Paramecium* genome allows the investigation of the fate of gene duplicates over different evolutionary scales.

The analysis of EST abundances suggested that in *Paramecium*, as in yeast, highly expressed genes tend to be more retained [17]. To investigate in detail the relation between gene expression and gene retention following WGD we measured genome-wide expression patterns in different culture conditions and at different stages of *Paramecium* life cycle. We show that retention rate is positively correlated with the level of gene expression. This observation does not appear to be due to indirect effects of other parameters known to affect gene retention. To explain these observations we propose a model based on the assumption that gene expression levels before WGD are close to an optimum, which corresponds to a trade-off between the benefit and cost of their expression. This simple COSTEX model provides a general explanation for the relationships between gene expression and gene evolution, not only in terms of gene dosage but also in terms of evolution of the encoded proteins.

## Results

### Expression level influences gene retention after WGD

We measured the expression level of *Paramecium* genes in 58 different experiments, spanning different stages of its life cycle, using a DNA microarray covering the 39,642 protein-coding genes annotated in the genome. We define here the expression level of a gene as the median value of its expression across all 58 different experiments. We name ‘ohnologon’ a set of ohnologous genes related by a given WGD event. Since the *Paramecium* lineage encountered 3 successive WGDs, ohnologons may contain from 1 up to 2, 4 or 8 genes for the recent, intermediary or old WGD respectively.

**Figure 1:**
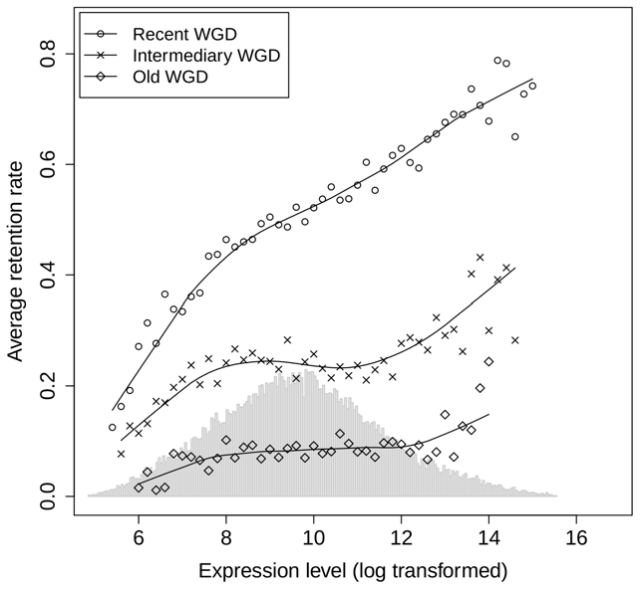
Relationship between gene expression level and the frequency of gene retention after WGDs. Ohnologons were binned according to their expression level, and for each bin, we computed the frequency of ohnologons having retained both copies since the WGD (see Materials and Methods). Circles: recent WGD (23,404 ohnologons); crosses: intermediary WGD (16,464 ohnologons); diamonds: old WGD (9,050 ohnologons). The histogram in the background represents the distribution of expression level for all genes in *Paramecium*. For each WGD the locally-weighted polynomial regression (lowess, as implemented in the R software [52]) is displayed as a solid line for visual aid.

Ideally, to investigate the relationship between gene expression and retention, one would have to measure the rate of gene loss per elementary time unit in each ohnologon. However, with only one genome sequenced in the *Paramecium* clade, it is not possible to quantify this rate for each individual ohnologon. We therefore investigated the relationship between gene expression and retention by grouping ohnologons into bins defined by fixed intervals of expression level (see Materials and Methods). For the recent WGD, there is a striking positive relationship between the frequency of gene retention in each bin and their average expression level (Figure 1). The frequency of gene retention increased 2-fold between the 10% least expressed genes and the 10% most highly expressed genes (0.32 and 0.67 respectively, P<10^−16^). We observed the same trend for the intermediary and the old WGD (frequency of retention = 0.17 vs. 0.31, P<10^−16^ and 0.04 vs. 0.10, P = 2.9×10^−6^ when comparing the 10% extreme genes respectively for the intermediary and old WGD). We also found a similar relationship between gene retention in the *Paramecium* lineage and the expression level of their orthologs in *Tetrahymena thermophila* (Figure S1). The divergence between *T. thermophila* and *P. tetraurelia* lineages occurred before the last two WGDs [17]. Hence, the observed correlation between expression level in *T. thermophila* and retention rate in *Paramecium* directly demonstrates that there is a relationship between the expression level of genes – before WGD – and their probability of retention after the WGD event. In other words, the selective pressure against gene losses is positively correlated to the pre-WGD expression level.

### Other factors contributing to gene retention

It has been shown that various parameters affect the fate of duplicated genes after WGD. Notably, some functional gene categories are more retained than others, possibly because they contributed to adaptation by functional innovation [11], or because of dosage balance constraints [16]–[18]. We analyzed each of the known factors in order to investigate whether the observed relationship between gene retention and expression could be explained by these other parameters.

### Gene retention versus phylogenetic distribution

It is expected that widely conserved genes and lineage-specific genes undergo different selective pressures [21], [22]. To investigate the relationship between retention rate and phylogenetic distribution, we classified genes into 3 groups: *Paramecium*-specific genes (*n* = 17,896), ciliate-specific genes (*n* = 4,135) and ancient eukaryotic genes (*n* = 8,846) (see Materials and Methods). We found that eukaryotic and ciliate-specific genes are more retained than average following the recent WGD (both *P*<10^−16^) while *Paramecium* specific genes were more frequently lost (*P*<10^−16^). Therefore, genes that are conserved across large evolutionary time scales are more prone to retention following WGD than genes that evolved quickly or were innovated in the *Paramecium* lineage. However, all 3 gene categories show a relationship between gene expression and gene retention similar to what we observed on the whole set of *Paramecium* genes (Figure S2), indicating that this relationship pertains independently of age or level of gene conservation.

### Gene retention versus functional categories

We classified *Paramecium* genes according to their functional category based on the Gene Ontology (GO) [23]. We computed the average retention rate for each functional category represented by more than 400 genes in the *Paramecium* genome. On average, genes that have a GO assignment are more retained than other genes (0.57 vs. 0.48, *P*<10^−16^). This result simply reflects the previous observation: given that functional category assignment is based on homology with genes in other species and that genes conserved across species are preferentially retained following WGD, genes with GO assignment tend to be more retained than the average. However, a few (3/23) functional categories were significantly under-retained (Table S1). Among them, ‘integral to membrane’ is the category with the lowest retention rate, reflecting differences in post-WGD selective pressure on genes encoding membrane proteins (see discussion).

We analyzed the relation between gene expression and gene retention across the different functional categories by dividing genes into 4 quartiles according to their expression level (Figure S3). As expected, functional categories show differences both in average expression levels and retention rates. For the same level of expression, different GO categories show different retention rates, which shows an effect of functional categories independently of gene expression. Nevertheless highly expressed genes (in the upper quartile) are more retained than lowly expressed ones (in the lower quartile) for all the 23 functional categories analyzed, indicating that the relationship between gene expression and retention is not caused by some specific functional categories (Figure S3 and Table S1).

### Gene retention versus dosage balance constraints

**Figure 2:**
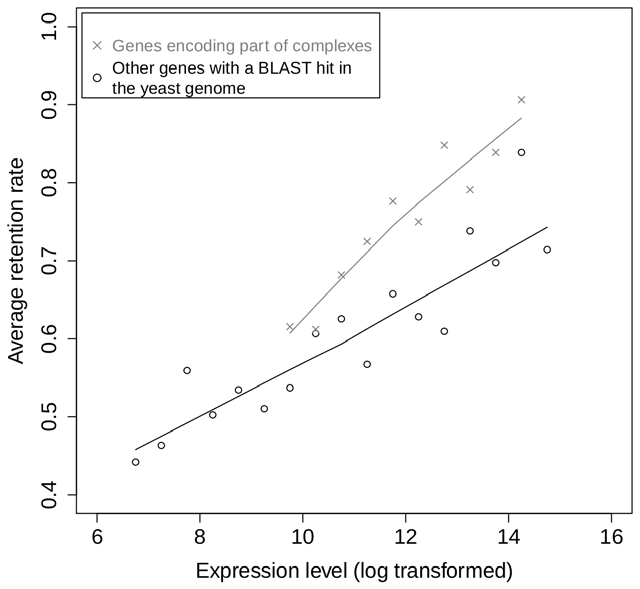
Relationship between gene expression and retention for subunits of protein complexes and for other genes. Retention rates were computed for bins of expression level for genes that are predicted to be involved in protein complexes by homology with yeast proteins (crosses) and for other genes having homologs in yeast (circles). The two sets contain respectively 590 and 4,384 ohnologons, grouped into 10 and 17 bins. The solid lines correspond to locally-weighted polynomial regression (lowess, as implemented in the R software [52]).

Aury et al. [17] showed that genes encoding subunits of protein complexes are over-retained after the recent WGD in *Paramecium*. We used the same data to investigate the relation between expression level and retention rate separately for genes predicted to encode part of protein complexes (*n* = 1,236) and for other genes (*n* = 7,025) (see Materials and Methods). We find that genes coding for subunits of protein complexes are over-retained, even when expression is controlled for (Figure 2), confirming the impact of dosage-balance constraints on the fate of genes following WGD. However, both genes encoding protein-complex subunits and other genes show a similar relationship between expression level and retention rate (Figure 2). Hence, expression level appears to influence the retention of genes following WGD, independently of dosage balance constraints.

### Highly expressed genes show no evidence of a higher tendency for change of function

Some duplicate genes are retained because they evolved toward different functions (by neo- or sub-functionalization) [11], [12]. One possible hypothesis to explain the higher retention of highly expressed genes is that they might be more prone to functional changes, either via changes in the encoded protein or via changes in expression patterns. To test this hypothesis, we first investigated the relation between gene expression and coding sequence divergence, measured by the rate of non-synonymous changes (*K_a_*) between ohnologs of the recent WGD. We found a negative correlation (*r* = −0.31, *P*<10^−16^; Figure S4), indicating that the evolutionary rate of coding sequences is lower in highly expressed genes.

We also investigated the relation between gene expression and the rate of evolution of expression patterns between ohnologs of the recent WGD. For this we used two different measures of expression divergence. The first is the Pearson correlation coefficient between ohnologs on the 58 different experiments. The second measure is an Euclidean distance between expression levels of ohnologous genes across the 58 different arrays. Both measures show a negative correlation between gene expression and divergence of expression patterns (*r* = −0.23 and *r* = −0.13 respectively, both *P*<10^−16^): highly expressed genes have more conserved expression patterns.

Thus, highly expressed genes evolve more slowly than weakly expressed genes, both in terms of protein sequence and in terms of expression pattern. These two observations are consistent with the model we propose (see discussion) but are in contradiction with the hypothesis that highly expressed genes undergo functional innovation more frequently than weakly expressed genes. We admit however that this latter hypothesis cannot be formally rejected. Indeed, it can be argued that functional innovations do not necessarily imply a noticeable increase in evolutionary rate (*e.g.* a very limited number of amino-acid changes might be sufficient to change the function of a protein), and the negative correlations reported above might reflect other evolutionary processes (*e.g.* selective constraints on amino-acid sequences to avoid protein folding errors [24]). The minimal conclusion is therefore that we found no evidence of a higher propensity for functional innovation among highly expressed genes.

## Discussion

### Gene expression and dosage sensitivity in *Paramecium*, yeast, and animals

We studied the constraints acting on the evolution of gene dosage by analyzing the fate of duplicated genes after WGDs. We show that the frequency of gene retention following the recent WGD in *Paramecium* is positively correlated to gene expression level, which reveals a selective pressure against the loss of highly expressed duplicated genes. Various factors are known to contribute to the retention of gene duplicates, such as a functional shift by neo or sub-functionalization, or selection for dosage balance in protein complexes. However, these factors do not appear to explain the observed relationship between retention rate and gene expression. Highly expressed genes do not show evidence of a higher propensity to evolve toward new functions after a duplication. Moreover, the relationship between retention rate and gene expression holds for most functional categories, independently of their involvement in protein complexes. Hence, the most parsimonious explanation for our observations is that there is a direct link between the expression level of genes and the fitness impact of changes in gene dosage.

**Figure 3:**
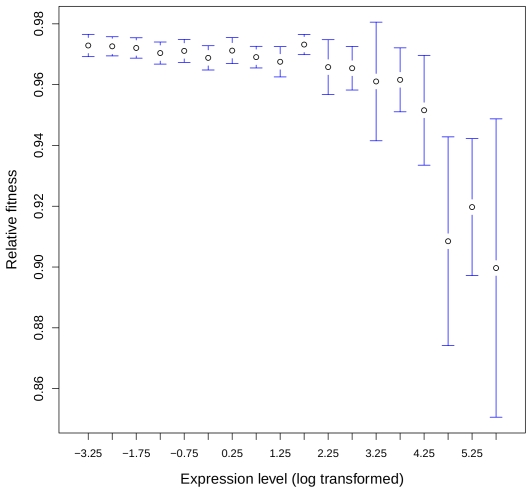
Relationship between gene expression level and loss of fitness associated to heterozygous KO in yeast. The fitness after deletion of one allele in yeast was taken as the minimal fitness measured across all conditions given in [25]. Genes were binned according to their expression level (expression data from [26], see Materials and Methods) and the average fitness computed for each bin. The 18 bins analyzed contained a total of 5,030 genes. Error bars correspond to the 95% confidence interval.

To test this hypothesis, we analyzed data from systematic gene knock-out (KO) experiments in the yeast *Saccharomyces cerevisiae*, where the fitness of heterozygous strains (*i.e.* carrying one KO allele and one wild-type allele) was measured by competition experiments [25], and for which expression data were available from [26]. We found a negative correlation between the fitness of heterozygotes and the expression level of the corresponding genes (*r* = −0.13, *P*<10^−16^). The mean loss of fitness increased 2-fold between the 10% least expressed genes and the 10% most highly expressed genes (0.027 and 0.053 respectively, P = 10^−10^; Figure 3) which indicates a higher selective pressure against reduction of gene dosage for highly expressed genes. Several observations suggest that this rule holds also for multicellular eukaryotes. First, *Drosophila* and mouse genes with copy number variation (CNVs), tend to be lowly expressed and/or have a narrow tissue distribution [27], [28]. Second, it is known that the small subset of genes on the human Y chromosome that have retained a homolog on the X chromosome is strongly biased toward highly expressed genes [29]. Both observations are consistent with the hypothesis that changes in gene dosage are more deleterious for highly expressed genes.

The strong correlation between gene expression and retention in *Paramecium* that is apparent in Figure 1 should not be interpreted as evidence that expression is the unique determinant of the variance in the rate of gene loss. Indeed, to analyze the relation between the frequency of gene loss in *Paramecium* and gene expression, we had to bin the data into groups of expression level. This binning tends to underestimate the variance between individual genes that is caused by other factors (*e.g.* see [30]). Thus, the strong correlations observed with binned data simply indicate that on average – everything else being equal – the fitness impact of gene loss is correlated with expression level, which does not exclude that other factors contribute to variations in retention rate.

### The COSTEX model: trade-off between benefit and cost of gene expression

It is clearly established that expression of a gene is a costly process, both because it requires energy (particularly for protein synthesis) and because it mobilizes cellular resources (*e.g.* the translational machinery), thus competing with the expression of other genes (see [31], [32] for a recent appraisal). Hence natural selection is expected to drive gene expression towards an optimum level at which the cost of increased expression is balanced by the resulting benefit on fitness. In some cases it has been possible to directly measure the cost of gene expression. For instance Dekel and Alon [32] measured the cost of gratuitous induction of the *lac* operon in *Escherichia coli*. They could also measure the fitness gain associated with *lac* induction as a function of available lactose concentration. Moreover, they showed by in-lab evolution experiments that optimal *lac* expression could be reached in just a few hundred generations, demonstrating the strength of selection for optimal gene expression. The selective pressure to optimize gene expression levels is expected to be particularly strong in microorganisms because of their large effective population sizes [31], but there is clear evidence for such selective pressures in animals too [33].

**Figure 4:**
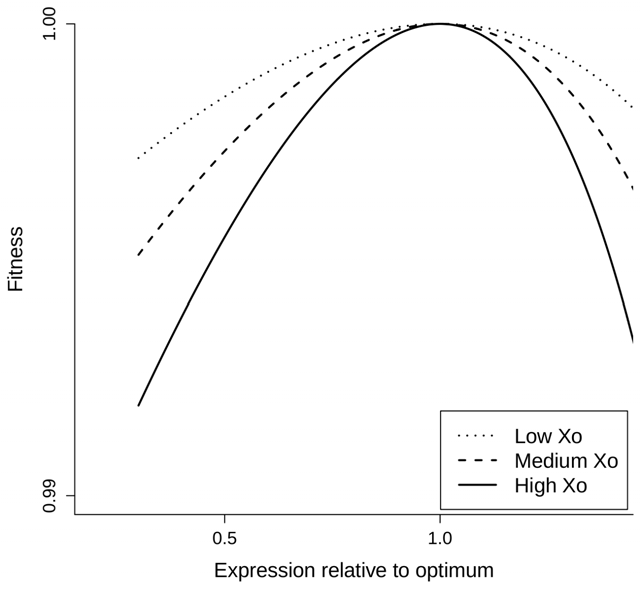
Fitness functions predicted by the COSTEX model for different values of optimal expression levels. These plots represent the fitness function *w*(*x*) for several values of *X_0_*. They were generated assuming an affine benefit function *B*(*x*) in equation (2) for increasing optimal expression levels *X_0_*: dotted, dashed and continuous lines for low, medium and high *X_0_*, respectively.

We now show that this selective pressure can explain the observed relationship between gene expression level and the fitness impact of changes in gene dosage. Our model is based on a simple cost function for gene expression in the presence of limiting resources that has been proposed by Dekel and Alon [32] on the basis of the Monod equation and that matched their data particularly well:
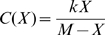
(1)where *X* is the gene expression level, *M* is the maximal capacity for expression of a gene, given the cellular resources that can be used for its expression and *k* is a scaling factor expressing the fitness cost of resource usage. Let *X_0_* be the optimal expression level of a gene, *i.e.* the level that maximizes fitness. We use the relative expression level *x* of this gene with respect to its optimal expression level: 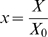. It should be noted that the optimal expression level of a given gene depends on resources available and therefore depends on the expression of all the other genes. Hence, *X_0_* for a given gene may change as the expression of other genes evolves. However, at equilibrium, selection should drive the evolution of expression levels of each gene close to a value that maximizes fitness (that is, *x* = 1). We express fitness *w*(*x*), a function of the relative gene expression level, as the difference between a benefit function *B*(*x*) and the cost function *C*(*X_0_x*):
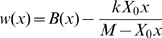
(2)Note that fitness is expressed relatively to the fitness of the optimal genotype (*i.e. X* = *X_0_*). Hence, fitness is equal to 1 for *x* = 1:
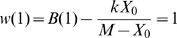
(3)For *x* = 1 the fitness function is also at an optimum, hence:
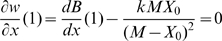
(4)so that 
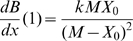
 is necessarily positive at optimal expression. Therefore *w*(*x*) can be approximated by a second order Taylor expansion:
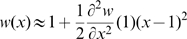
(5)Therefore the selective pressure on changes in relative expression level *x* can be quantified by the magnitude of the second order derivative:
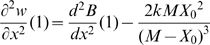
(6)which must be negative at maximal fitness. Therefore, everything else being equal, the selective pressure on relative gene expression level is predicted to increase with the optimal expression level *X_0_*. This is illustrated on Figure 4 showing the fitness function *w*(*x*) for various values of *X_0_* assuming an affine benefit function *B*(*x*). The higher the optimal expression level *X_0_*, the sharper the fitness function is in the vicinity of this optimum – equation (6) – resulting in increased selective pressure on gene expression.

As a first approximation, the loss of a gene copy after WGD is expected to decrease by 50% the level of gene expression. Under the assumption that most genes were close to their optimal expression at the time of WGD, we can estimate the selection coefficient *s* associated with the drop in expression following the loss of an ohnolog by setting 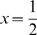 in equations (5) and (6):
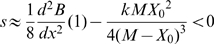
(7)This approximation by Taylor expansion is all the more accurate as *X*_0_ is low compared to *M*. This relationship predicts that the strength of selection against gene loss increases with gene expression, as observed very clearly in the present work for the recent *Paramecium* WGD (Figure 1). On longer time scales, other processes such as neo- or sub-functionalization are expected to contribute to gene retention, which may explain why the relationship between retention rate and expression level is weaker for the intermediary and old WGDs (Figure 1).

### The COSTEX model and the evolutionary path to pseudogenization

On shorter time scales, an additional phenomenon may contribute to the selective pressure against loss of highly expressed genes. Indeed, gene losses are usually caused by the accumulation of small-scale mutational events [17], transiently resulting in the expression of a non-functional peptide. Disabling mutations that disrupt the function of the protein but do not change its expression level clearly bear a cost with no benefit. The corresponding selection coefficient *s_ψ_* can be derived from equations (2) and (4) at 1^st^ order approximation:
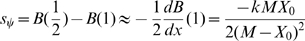
(8)This cost may even be higher if the non-functional peptide interacts with other proteins and perturbs their functions in a dominant-negative fashion, so that 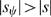 is a lower bound for the selection coefficient. Therefore the COSTEX model predicts that gene expression strongly influences the pseudogenization path to gene loss because the probability of fixation of disabling mutations decreases with increasing gene expression level. Moreover this model predicts that once a disabling mutation has been fixed, there should be a selective pressure to decrease the expression level of the pseudogene up to its total silencing, all the stronger as gene expression is high.

### The COSTEX model: gene-specific parameters

Gene expression level is obviously not the unique determinant of gene evolution. As shown in equation 6, there are several other parameters that determine the selective pressure against changes in gene dosage. First, parameters *M* and *k* of the cost function are expected to vary from one gene to another, according to the length of encoded proteins and their amino-acid composition. Moreover, the amount of resources available for gene expression depends on the physiological state of the cell, and hence these parameters should also depend on the time at which genes are expressed. Second, the selective pressure against changes in gene expression also depends on the second derivative of the benefit function *B(x)* (see equations 5–7). Little is known about the shape of the benefit function – except that this function must be increasing in the vicinity of the optimal expression level (see equation 4). It is however clear that *B(x)* certainly varies widely among genes. Indeed, it is well known that there are some weakly expressed genes that are essential for cell functioning (*e.g.* transcription factors). In other words, the fact that the optimal expression of a gene is low does not necessarily imply that the fitness impact of mutations affecting its expression is low.

Thus, the selection coefficient against changes in gene expression *s* is expected to vary according to gene-specific parameters 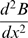, *k* and *M*. We observed indeed that for a same expression level, the frequency of gene retention among *Paramecium* ohnologs varies strongly according to functional GO categories (Figure S3). In absence of knowledge about these parameters it is difficult to predict *s* for any given gene. However, under the assumption that the distribution of these parameters is similar among genes of different expression levels, the COSTEX model predicts that, on average, selective constraints on gene dosage increase with expression level.

### Gene expression optimality after WGD

The COSTEX model can explain the observed relationship between gene retention rate and expression level, under the assumption that most genes were close to their optimal expression level right after WGD. This hypothesis is difficult to test but deserves to be discussed because it is a major assumption of the model. In the absence of major changes such as WGDs, most genomes are expected to tend toward this evolutionary equilibrium at which most genes are expressed close to their optimum level [33]. Therefore, the ancestral pre-duplication species in the *Paramecium* lineage was probably in this situation. The question now turns into: how did the WGD affect this equilibrium? A first point to note is that in-lab polyploidisation experiments in plants and yeast indicate that changing the ploidy from 2n to 4n has very little influence *per se* on the relative expression level of genes [34]–[36]. Such experiments showed that allopolyploidization (*i.e.* WGD resulting from inter-species hybridization) affects the expression of many more genes than autopolyploidization, and that these changes can have very important phenotypic consequences [37]. However, even in the case of allopolyploidization, a large majority of genes do not show substantial changes of expression level relative to the parental species (*e.g.* in *Arabidopsis* allotetraploids, less than 10% of genes show a 1.5-fold difference in gene expression [35]). Second, the relative dosage between genes remains unchanged until gene losses start to accumulate. Third, it has been observed, both in plants and in yeasts, that cell size increases with the level of ploidy [34], [38], [39]. These three points suggest that a WGD event does not necessarily result in a change in the concentration of cytoplasmic proteins. It should be noted however that, when the volume of a cell increases, the surface of its membrane should increase in a lower proportion, and hence the surface concentration of membrane proteins might be too high immediately after WGD. This could explain our observation that genes encoding membrane proteins are under-retained. However, in the specific case of *Paramecium*, the relation between ploidy and cell volume is unclear because of nuclear dimorphism. *Paramecium*, like other ciliates, separates germline and somatic functions into two distinct nuclei (named respectively micronucleus and macronucleus). The transcriptionally silent micronucleus is diploid while the expressed macronucleus is highly polyploid (∼800 *n*). WGDs resulted in a temporary tetraploidization of the micronucleus but one can only speculate about the consequences on macronucleus ploidy. Indeed, it has been shown that the macronucleus DNA content is regulated after amitotic divisions [40], leaving open the possibility that micronucleus tetraploidization did not change the total amount of DNA in the macronucleus.

Although we can only speculate on the immediate consequences of WGD in *Paramecium*, it can be argued that the fixation of a WGD in the population of ancestral species would be highly unlikely if it resulted in a strong decrease in fitness. This is particularly true in microorganisms such as *Paramecium* for which selection against fixation of deleterious mutations is strong because of their high effective population size [41]. Therefore, assuming that expression level of most genes was close to their optimum immediately after WGD appears to be a reasonable assumption.

### The trade-off between cost and benefit of gene expression constrains evolutionary rates of coding sequences

One additional prediction of the COSTEX model is that the selective constraints on coding sequences should vary with gene expression level. Indeed, missense mutations in a coding sequence do not change expression level (and therefore do not change the cost of expression), but they generally yield a decrease of the benefit function. Hence, the fitness function for a mutant allele becomes (see equation 2):
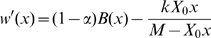
(9)where α denotes the decrease of the benefit function caused by this particular allele, and *x* and *X_0_* correspond to the expression parameters of the wild-type allele. Therefore the effect of the missense mutation on fitness is:
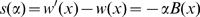
(10)If the wild-type gene was at its optimal expression level (*x* = 1), *B*(1) can be inferred from equation (3), which leads to:
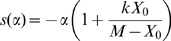
(11)which indicates that the loss of fitness is an increasing function of gene expression. Hence mutations with an equivalent effect on protein function are predicted to have a stronger impact on fitness for highly expressed genes because of the higher cost incurred for their expression, a price the organism had to ‘pay’ for their function. Note that this relationship also applies for potentially suboptimal expression 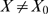. Note also that the distribution of α for the different mutations that may affect a gene probably differs widely from gene to gene. In other words, there are some genes for which, on average, mutations have a stronger impact on their benefit function than others. Hence, the mean fitness impact of mutations depends not only on *X_0_*, but also on the distribution of α, which is gene-specific. Therefore this model does not contradict the observation that some lowly expressed proteins may also be under strong selective constraints. Nevertheless, under the null hypothesis that the distribution of α is independent of the level of gene expression, the COSTEX model predicts that, on average, the selective constraints on coding sequences are higher in highly expressed genes.

### Conclusion

It is well established that the expression pattern of genes is an important determinant of the rate of evolution of the encoded proteins [42], [43], although the reasons for this observation are still debated (for review, see [44]). Here we show that gene expression is also a major determinant of the evolution of gene dosage. Thus, many aspects of gene evolution appear to be driven by constraints on gene expression. To explain the observed relationship between gene expression level and the fitness impact of both changes in gene expression and changes in the encoded protein, we propose a model, based on the simple assumption that gene expression levels reflect a trade-off between cost and benefit of gene expression. This model is directly inspired by the work by Dekel and Alon who demonstrated and quantified experimentally the cost of gene expression *in vivo* [32]. Put in a simple verbal formulation, the COSTEX model states that because of the non-linearity of the cost function, gene evolution (in terms of gene expression, gene dosage or encoded proteins) is all the more constrained as optimal gene expression is high. Thus this model can explain simultaneously three observations in *Paramecium*: i) highly expressed genes are more frequently retained as duplicates after a WGD, ii) they evolve more slowly than other genes in terms of protein divergence and iii) they evolve more slowly than other genes in terms of expression pattern. Note that the COSTEX model does not imply that gene expression is the unique determinant of gene evolution. Selective constraints notably depend on the shape of the benefit function, which certainly varies widely among genes. However, the COSTEX model can explain why, on average, highly expressed genes are more constrained than others.

Several other hypotheses have been proposed to explain the relationship between gene expression and the rate of protein evolution [44]. According to a popular model, this relationship reflects a selective pressure on protein sequences to prevent folding errors [24]. Indeed, misfolded proteins can affect fitness, either directly (they can be toxic for the cell) or indirectly (they represent a waste of resources). In both cases the impact on fitness is dependent on gene expression level, and hence this model predicts a stronger selective pressure on highly expressed protein-coding sequences. Translational errors represent one important cause of protein misfolding [45]. Thus, one interesting feature of this model is that it provides an explanation for the covariation between codon usage (under selection to optimize translation accuracy) and non-synonymous substitution rate [24]. The ‘misfolding hypothesis’ and the COSTEX model are not mutually exclusive. In fact, the waste of resources linked to the production and degradation of misfolded proteins can be considered as one component of the cost of gene expression. But the COSTEX model predicts that even in absence of folding errors, the rate of protein evolution should be negatively correlated to the expression level. One other interesting aspect of the COSTEX model is that it also provides an explanation for the relationship between gene expression and the evolution of gene dosage or gene expression, an aspect of gene evolution that is not predicted by the ‘misfolding hypothesis’. Thus, the COSTEX model provides a simple and common explanation for the general relationship observed between the level of gene expression and the different facets of gene evolution.

## Materials and Methods

### Expression data

Expression data for *P. tetraurelia* were obtained from single channel NimbleGen arrays with six different 50-mer probes per gene. We analyzed data from a total of 58 different hybridizations, corresponding to six independent series of experiments (raw data are deposited in the Gene Expression Omnibus database [46], under accession numbers GSE18002, GSE17998, GSE17997, GSE17996, GSE17930, GSE14631 and GSE12620). Signals from the 58 arrays were simultaneously normalized using the normalizeBetweenArrays function from the Limma package [47]. The expression of each gene in each condition was taken as the median of the six individual 50-mer signals. We calculated expression level of each gene as the log2 of the median value across all 58 arrays. Expression levels of ohnologons were taken as that of a randomly chosen gene within each ohnologon [17], [48].

Ohnologons were sorted according to their expression level and grouped into bins defined by fixed intervals of expression level. Depending on the size of the dataset, this interval was set to 0.2 or to 1. Bins containing less than 30 ohnologons were excluded from the analysis. Retention rate was calculated in each bin as the frequency of ohnologons having retained both gene copies.

Microarray data for *T. thermophila* [49] were downloaded from the Gene Expression Omnibus (http://www.ncbi.nlm.nih.gov/geo/), a public repository of expression data [46]. We normalized data across all 50 available arrays (GEO series: GSE11300) and computed expression level of each gene as the median value across all 50 arrays. Orthology relationships between *P. tetraurelia* and *T. thermophila* were taken from [17].

### Functional categories

Functional categories were downloaded from parameciumDB (http://paramecium.cgm.cnrs-gif.fr/download/analysis/InterproScan_results_August_2008.txt) and only categories with more than 400 genes were retained. We eliminated redundancy among functional categories by searching for categories for which both gene lists overlapped by more than 90%. In these cases the category with the higher number of assigned genes was retained. This led to the elimination of three functional categories: protein kinase activity (GO:4672), protein serine/threonine kinase activity (GO:4674) and ribosome (GO:5840), that overlapped protein amino acid phosphorylation (GO:6468), protein kinase activity (GO:4672) and structural constituent of ribosome (GO:3735), respectively. Each functional category was divided into 4 bins of equal size according to gene expression level and we computed average retention rates for each quartile.

### Phylogenetic distribution

Lists of orthologous genes were obtained through the BioMart interface of parameciumDB [50]. For *Paramecium* specific genes we queried the BioMart interface for all *Paramecium* genes with no ortholog in any other species available. Ciliate-specific genes were obtained by querying for genes with an ortholog in *T. thermophila* only and ancient eukaryotic genes by querying for genes with an ortholog in *H. sapiens*.

### Proteins involved in complexes

Paramecium genes encoding subunits of protein complexes were predicted by Aury and colleagues [17] by homology with yeast proteins annotated in the MIPS database (http://mips.gsf.de/) or in [51]. The rate of retention is also correlated to the level of conservation of genes across the eukaryote phylogeny (see text). In order to investigate the impact of protein complexes on the rate of gene retention independently of their phylogenetic distribution, we selected a set of *Paramecium* genes having an homolog in yeast (defined as genes having at least one BLASTP hit in the yeast proteome with *P*<1×10^−3^ and alignment covering >70% of the *Paramecium* protein) and compared retention rates for genes involved in protein complexes (*n* = 615 ohnologons) and for other genes (*n* = 4,331 ohnologons).

### Yeast KO data

We defined the fitness associated to a heterozygous KO as the minimal fitness across the different culture conditions tested in [25]. Expression level for each gene corresponds to the log2-transformed value of mRNA abundance per cell given by [26].

## Supporting Information

Figure S1 Relationship between the rate of gene retention in the *Paramecium* lineage and the expression level of their orthologs in *T. thermophila*. Ohnologons were binned according to expression levels of their orthologs in *T. thermophila*, and for each bin, we computed the frequency of ohnologons having retained both copies since the WGD. Circles: recent WGD (3,601 ohnologons); crosses: intermediary WGD (2,998 ohnologons); diamonds: old WGD (1,589 ohnologons). The histogram in the background represents the distribution of expression levels in *Tetrahymena* for genes that have an ortholog in *Paramecium*. For each WGD the locally-weighted polynomial regression (lowess, as implemented in R [52]) is displayed as a solid line for visual aid. For the recent and the intermediary WGDs the frequency of gene retention significantly increased between the 10% least expressed genes and the 10% most highly expressed genes (0.49 vs. 0.84, P<10^−16^ for the recent WGD and 0.24 vs. 0.48 P = 2.6×10^−10^ for the intermediary WGD) while it was not significant for the ancient WGD (0.16 vs. 0.19, P = 0.37).
(2.68 MB TIF)

Figure S2 Relationship between gene expression and gene retention for genes with different phylogenetic distributions. Retention rates after the recent WGD were computed for bins of expression level for genes that are *Paramecium*-specific (*n* = 10,861 ohnologons), ciliate-specific (*n* = 2,417 ohnologons) or ancient eukaryotic genes (*n* = 5,048 ohnologons) (see Materials and Methods). The horizontal dashed line represents the average retention rate following the recent WGD. The solid lines correspond to locally-weighted polynomial regression (lowess, as implemented in the R software [52]).
(6.34 MB TIF)

Figure S3 Relationship between gene expression and gene retention across different functional categories. Functional categories were taken from the Gene Ontology classification [53] as indicated in each panel. For each category, ohnologons were grouped into four quartiles of expression level and the average retention rate was computed as the frequency of ohnologons having retained both copies since the recent WGD. The dotted line corresponds to the average retention rate of all genes with a GO classification.
(3.15 MB TIF)

Figure S4 Relationship between non-synonymous substitution rates and expression level. Values of non-synonymous divergence (Ka) between ohnologs from the recent WGD were taken from [17]. The solid red line shows the linear regression between Ka and expression level.
(2.56 MB TIF)

Table S1 Detailed analysis of functional categories. For each functional category, the indications given by the table are:
*go*: GO number of the functional category.
*name*: name of the functional category.
*type*: type of functional category (‘Molecular function’, ‘Biological process’ or ‘Molecular function’).
*nbg*: the number of genes within a given functional category.
*retention*: the average retention rate among genes belonging to the functional category.
*retention*_*others* : the average retention of genes not belonging to the given GO category.
*pval*_*retentions*: p-value associated to the comparison of the 2 retention rates by a Chi2 test (bold when <0.05; grey background when retention rate is lower than other genes).
*avg_xp*: average expression level of genes belonging to the functional category.
*avg_xp_others*: average expression level of genes not belonging to the functional category.
*pval_xp*: *p*-value associated to the comparison of the 2 average expression levels by a student t-test (bold when P<0.05; grey background when average expression level is lower than other genes).
*retention_quartile*#1–4: average retention rate among genes from each quartile of expression level (quartile#1 = low expression level; quartile#4 = high expression level).
*avg_xp_quartile*#1–4: average expression level in each quartile.
(0.01 MB PDF)
