# Correction: The Relationship among Gene Expression, the Evolution of Gene Dosage, and the Rate of Protein Evolution

**DOI:** 10.1371/annotation/c55d5089-ba2f-449d-8696-2bc8395978db

**Published:** 2010-06-08

**Authors:** Jean-François Gout, Daniel Kahn, Laurent Duret, Paramecium Post-Genomics Consortium

The first four columns in Table S1 were missing (values given in the table remain unchanged). The correct version of Table S1 can be viewed here: 
