# Supplementary figures and images for: The Relationship among Gene Expression, the Evolution of Gene Dosage, and the Rate of Protein Evolution
Source: PLoS Genet. 2010 May 13;6(5):e1000944. doi: 10.1371/journal.pgen.1000944 (PMC2869310; doi:10.1371/journal.pgen.1000944)

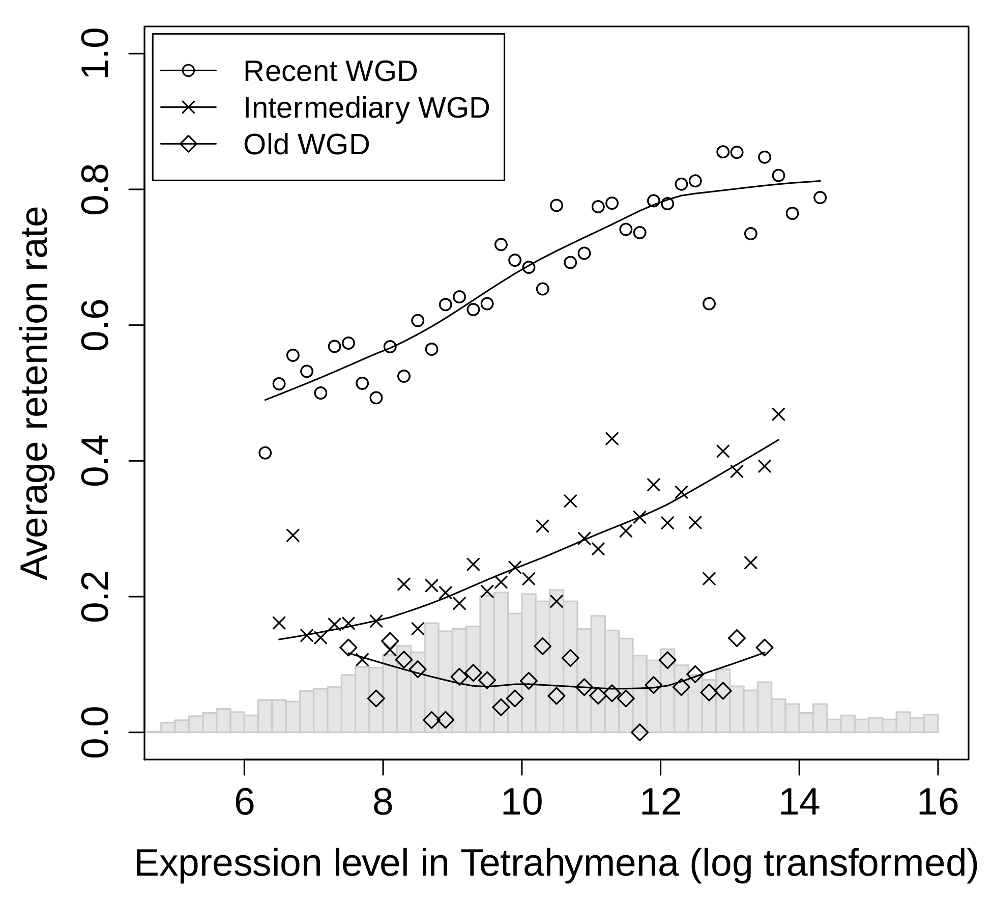

Supplement: Figure S1 — Relationship between the rate of gene retention in the Paramecium lineage and the expression level of their orthologs in T. thermophila. Ohnologons were binned according to expression levels of their orthologs in T. thermophila, and for each bin, we computed the frequency of ohnologons having retained both copies since the WGD. Circles: recent WGD (3,601 ohnologons); crosses: intermediary WGD (2,998 ohnologons); diamonds: old WGD (1,589 ohnologons). The histogram in the background represents the distribution of expression levels in Tetrahymena for genes that have an ortholog in Paramecium. For each WGD the locally-weighted polynomial regression (lowess, as implemented in R [52]) is displayed as a solid line for visual aid. For the recent and the intermediary WGDs the frequency of gene retention significantly increased between the 10% least expressed genes and the 10% most highly expressed genes (0.49 vs. 0.84, P<10−16 for the recent WGD and 0.24 vs. 0.48 P = 2.6×10^−10^ for the intermediary WGD) while it was not significant for the ancient WGD (0.16 vs. 0.19, P = 0.37). (2.68 MB TIF) [file pgen.1000944.s001.tif]

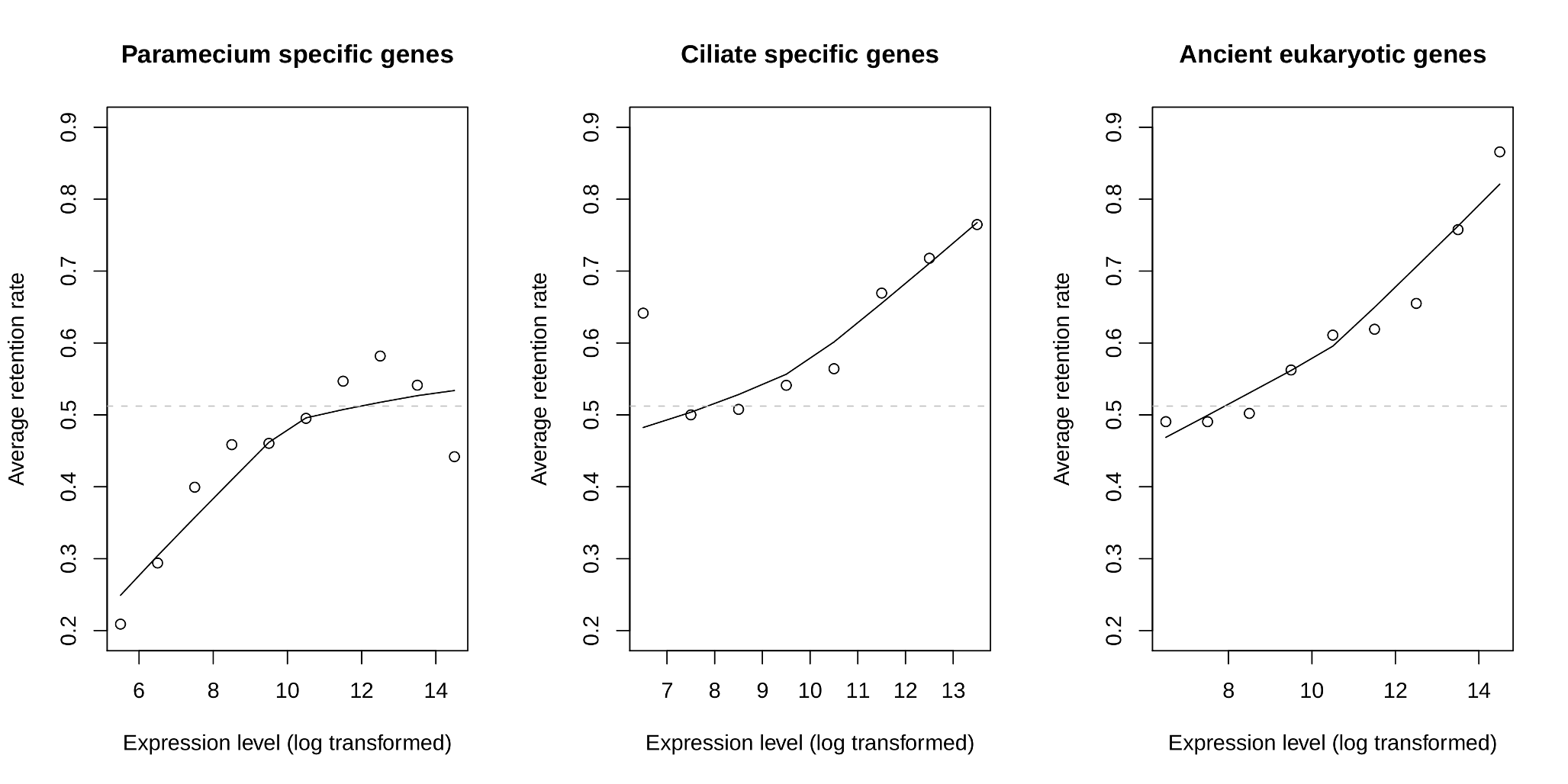

Supplement: Figure S2 — Relationship between gene expression and gene retention for genes with different phylogenetic distributions. Retention rates after the recent WGD were computed for bins of expression level for genes that are Paramecium-specific (n = 10,861 ohnologons), ciliate-specific (n = 2,417 ohnologons) or ancient eukaryotic genes (n = 5,048 ohnologons) (see Materials and Methods). The horizontal dashed line represents the average retention rate following the recent WGD. The solid lines correspond to locally-weighted polynomial regression (lowess, as implemented in the R software [52]). (6.34 MB TIF) [file pgen.1000944.s002.tif]

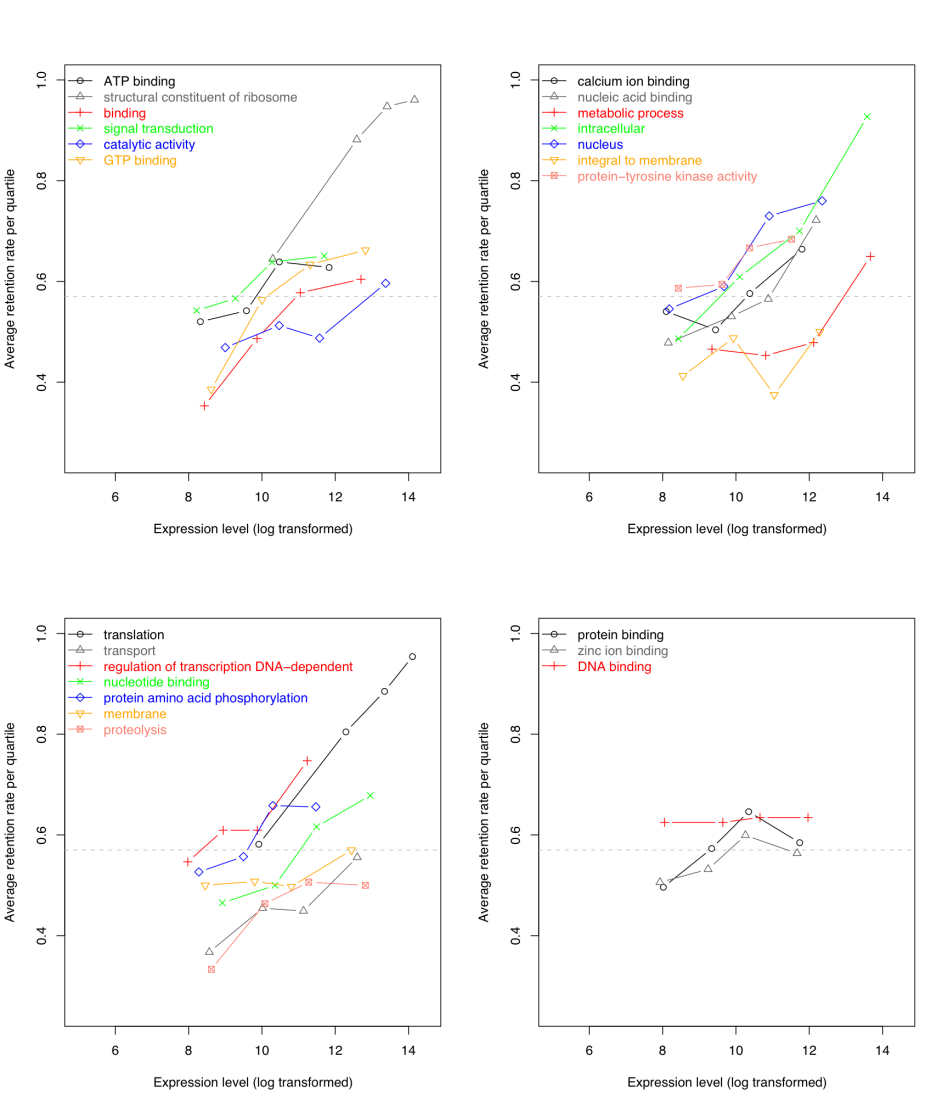

Supplement: Figure S3 — Relationship between gene expression and gene retention across different functional categories. Functional categories were taken from the Gene Ontology classification [53] as indicated in each panel. For each category, ohnologons were grouped into four quartiles of expression level and the average retention rate was computed as the frequency of ohnologons having retained both copies since the recent WGD. The dotted line corresponds to the average retention rate of all genes with a GO classification. (3.15 MB TIF) [file pgen.1000944.s003.tif]

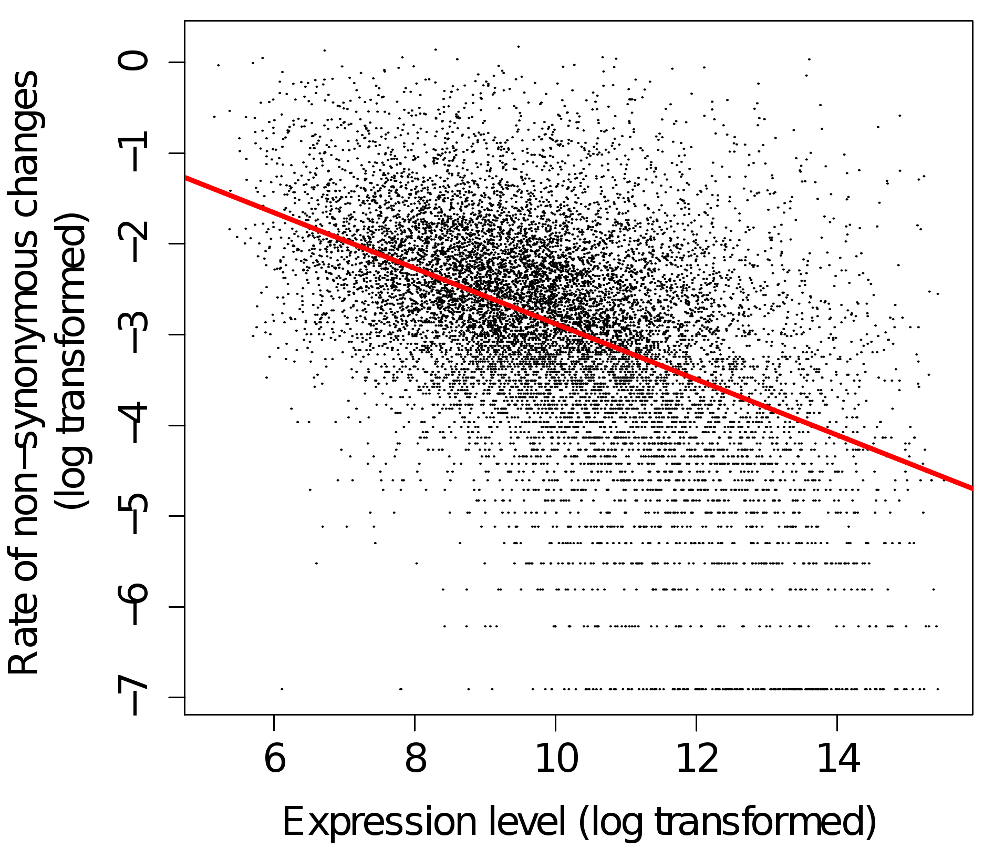

Supplement: Figure S4 — Relationship between non-synonymous substitution rates and expression level. Values of non-synonymous divergence (Ka) between ohnologs from the recent WGD were taken from [17]. The solid red line shows the linear regression between Ka and expression level. (2.56 MB TIF) [file pgen.1000944.s004.tif]
