# Supplementary material for: The Relationship among Gene Expression, the Evolution of Gene Dosage, and the Rate of Protein Evolution
Source: PLoS Genet. 2010 May 13;6(5):e1000944. doi: 10.1371/journal.pgen.1000944 (PMC2869310; doi:10.1371/journal.pgen.1000944)
Supplement: Table S1 — Detailed analysis of functional categories. For each functional category, the indications given by the table are: go: GO number of the functional category. name: name of the functional category. type: type of functional category (‘Molecular function’, ‘Biological process’ or ‘Molecular function’). nbg: the number of genes within a given functional category. retention: the average retention rate among genes belonging to the functional category. retention_others : the average retention of genes not belonging to the given GO category. pval_retentions: p-value associated to the comparison of the 2 retention rates by a Chi2 test (bold when <0.05; grey background when retention rate is lower than other genes). avg_xp: average expression level of genes belonging to the functional category. avg_xp_others: average expression level of genes not belonging to the functional category. pval_xp: p-value associated to the comparison of the 2 average expression levels by a student t-test (bold when P<0.05; grey background when average expression level is lower than other genes). retention_quartile#1–4: average retention rate among genes from each quartile of expression level (quartile#1 = low expression level; quartile#4 = high expression level). avg_xp_quartile#1–4: average expression level in each quartile. (0.01 MB PDF) [file pgen.1000944.s005.pdf]

retention rate	average retention rate of other genes	pval retentions	Average expression	Average expression in the rest of the genome	pval expr	retention quartile #1	retention quartile #2	retention quartile #3	retention quartile #4	Average expression in quartile #1	Average expression in quartile #2	Average expression in quartile #3	Average expression in quartile #4	r
0,58	0,50	5,38E-13	10,09	9,64	1,03E-38	0,52	0,54	0,64	0,63	8,32	9,58	10,48	11,83	0,87
0,60	0,51	7,30E-13	9,89	9,67	1,77E-09	0,53	0,56	0,66	0,66	8,28	9,50	10,30	11,47	0,91
0,63	0,51	1,89E-18	10,00	9,67	3,29E-16	0,59	0,59	0,67	0,68	8,43	9,62	10,37	11,52	0,92
0,58	0,51	3,45E-05	9,87	9,68	1,59E-04	0,50	0,57	0,65	0,58	8,02	9,34	10,35	11,74	0,68
0,55	0,51	1,39E-02	9,78	9,68	5,35E-02	0,51	0,53	0,60	0,56	7,93	9,24	10,26	11,67	0,73
0,68	0,51	2,22E-24	10,94	9,64	1,09E-63	0,49	0,61	0,70	0,93	8,43	10,10	11,73	13,58	0,99
0,51	0,51	7,01E-01	10,49	9,66	2,54E-31	0,35	0,49	0,58	0,60	8,43	9,87	11,05	12,70	0,95
0,52	0,51	7,11E-01	10,42	9,66	5,18E-32	0,50	0,51	0,50	0,57	8,45	9,80	10,80	12,44	0,80
0,52	0,51	8,32E-01	11,10	9,65	2,76E-72	0,47	0,51	0,49	0,60	9,00	10,47	11,57	13,37	0,87
0,57	0,51	7,80E-03	9,95	9,68	8,99E-05	0,54	0,50	0,58	0,66	8,10	9,44	10,38	11,80	0,83
0,57	0,51	7,53E-03	10,30	9,68	1,26E-13	0,48	0,53	0,57	0,72	8,15	9,88	10,88	12,19	0,93
0,51	0,51	9,84E-01	11,45	9,65	1,68E-72	0,47	0,45	0,48	0,65	9,35	10,81	12,12	13,67	0,82
0,63	0,51	1,30E-06	10,06	9,68	3,09E-06	0,63	0,63	0,63	0,63	8,05	9,64	10,66	11,97	0,86
0,66	0,51	5,88E-09	10,28	9,68	4,30E-11	0,55	0,59	0,73	0,76	8,18	9,67	10,91	12,35	0,96
0,81	0,51	1,88E-28	12,38	9,65	2,37E-89	0,58	0,80	0,89	0,95	9,91	12,29	13,35	14,11	1,00
0,46	0,51	2,65E-02	10,62	9,67	9,55E-24	0,37	0,45	0,45	0,56	8,56	10,02	11,13	12,60	0,95
0,63	0,51	1,33E-05	9,53	9,69	3,87E-02	0,55	0,61	0,61	0,75	7,98	8,94	9,87	11,24	0,95
0,57	0,51	4,74E-02	10,94	9,67	3,05E-35	0,47	0,50	0,62	0,68	8,92	10,36	11,49	12,95	0,97
0,86	0,51	5,16E-34	12,57	9,65	2,06E-89	0,64	0,88	0,95	0,96	10,30	12,59	13,41	14,16	0,98
0,60	0,51	1,38E-03	9,92	9,68	4,61E-03	0,54	0,57	0,64	0,65	8,22	9,27	10,28	11,70	0,94
0,45	0,51	2,57E-02	10,71	9,67	2,52E-22	0,33	0,46	0,51	0,50	8,62	10,08	11,28	12,82	0,87
0,44	0,51	1,36E-02	10,45	9,68	4,68E-16	0,41	0,49	0,38	0,50	8,55	9,93	11,04	12,29	0,35
0,56	0,51	9,31E-02	10,73	9,68	5,88E-18	0,39	0,56	0,63	0,66	8,62	10,00	11,31	12,82	0,93
