# Supplementary material for: Correction: The Relationship among Gene Expression, the Evolution of Gene Dosage, and the Rate of Protein Evolution
Source: PLoS Genet. 2010 Jun 8;6(6):10.1371/annotation/c55d5089-ba2f-449d-8696-2bc8395978db. doi: 10.1371/annotation/c55d5089-ba2f-449d-8696-2bc8395978db (PMC2890702; doi:10.1371/annotation/c55d5089-ba2f-449d-8696-2bc8395978db)
Supplement: Supplementary file 1 [file pgen.c55d5089-ba2f-449d-8696-2bc8395978db.s001.pdf]

go	name	type	number of genes	retention rate	average retention rate of other genes	pval retentions	Average expression	Average expression in the rest of the genome	pval expr	retention quartile #1	retention quartile #2	retention quartile #3	retention quartile #4	Average expression in quartile #1	Average expression in quartile #2	Average expression in quartile #3	Average expression in quartile #4	r
5524	ATP binding	Molecular Function	4077	0,58	0,50	5,38E-13	10,09	9,64	1,03E-38	0,52007	0,54181	0,6388	0,62771	8,32	9,58	10,48	11,83	0,87
6468	protein amino acid phosphorylation	Biological Process	2718	0,60	0,51	7,30E-13	9,89	9,67	1,77E-09	0,52658	0,55696	0,65823	0,6557	8,28	9,50	10,30	11,47	0,91
4713	protein-tyrosine kinase activity	Molecular Function	2177	0,63	0,51	1,89E-18	10,00	9,67	3,29E-16	0,58654	0,59425	0,66667	0,68371	8,43	9,62	10,37	11,52	0,92
5515	protein binding	Molecular Function	1762	0,58	0,51	3,45E-05	9,87	9,68	1,59E-04	0,49615	0,57308	0,64615	0,58462	8,02	9,34	10,35	11,74	0,68
8270	zinc ion binding	Molecular Function	1690	0,55	0,51	1,39E-02	9,78	9,68	5,35E-02	0,50598	0,53175	0,59921	0,56349	7,93	9,24	10,26	11,67	0,73
5622	intracellular	Cellular Component	1557	0,68	0,51	2,22E-24	10,94	9,64	1,09E-63	0,48636	0,60909	0,7	0,92727	8,43	10,10	11,73	13,58	0,99
5488	binding	Molecular Function	1325	0,51	0,51	7,01E-01	10,49	9,66	2,54E-31	0,35294	0,48663	0,57754	0,60428	8,43	9,87	11,05	12,70	0,95
16020	membrane	Cellular Component	1290	0,52	0,51	7,11E-01	10,42	9,66	5,18E-32	0,5	0,50777	0,49741	0,56995	8,45	9,80	10,80	12,44	0,80
3824	catalytic activity	Molecular Function	1040	0,52	0,51	8,32E-01	11,10	9,65	2,76E-72	0,46875	0,5125	0,4875	0,59627	9,00	10,47	11,57	13,37	0,87
5509	calcium ion binding	Molecular Function	834	0,57	0,51	7,80E-03	9,95	9,68	8,99E-05	0,54032	0,504	0,576	0,664	8,10	9,44	10,38	11,80	0,83
3676	nucleic acid binding	Molecular Function	784	0,57	0,51	7,53E-03	10,30	9,68	1,26E-13	0,47826	0,53043	0,56522	0,72174	8,15	9,88	10,88	12,19	0,93
8152	metabolic process	Biological Process	746	0,51	0,51	9,84E-01	11,45	9,65	1,68E-72	0,46552	0,45299	0,47863	0,64957	9,35	10,81	12,12	13,67	0,82
3677	DNA binding	Molecular Function	719	0,63	0,51	1,30E-06	10,06	9,68	3,09E-06	0,625	0,625	0,63462	0,63462	8,05	9,64	10,66	11,97	0,86
5634	nucleus	Cellular Component	705	0,66	0,51	5,88E-09	10,28	9,68	4,30E-11	0,54545	0,59	0,73	0,76	8,18	9,67	10,91	12,35	0,96
6412	translation	Biological Process	641	0,81	0,51	1,88E-28	12,38	9,65	2,37E-89	0,5814	0,8046	0,88506	0,95402	9,91	12,29	13,35	14,11	1,00
6810	transport	Biological Process	617	0,46	0,51	2,65E-02	10,62	9,67	9,55E-24	0,36735	0,45455	0,44898	0,55556	8,56	10,02	11,13	12,60	0,95
6355	regulation of transcription DNA-dependent	Biological Process	613	0,63	0,51	1,33E-05	9,53	9,69	3,87E-02	0,54651	0,6092	0,6092	0,74713	7,98	8,94	9,87	11,24	0,95
166	nucleotide binding	Molecular Function	582	0,57	0,51	4,74E-02	10,94	9,67	3,05E-35	0,46512	0,5	0,61628	0,67816	8,92	10,36	11,49	12,95	0,97
3735	structural constituent of ribosome	Molecular Function	574	0,86	0,51	5,16E-34	12,57	9,65	2,06E-89	0,64474	0,88158	0,94737	0,96053	10,30	12,59	13,41	14,16	0,98
7165	signal transduction	Biological Process	567	0,60	0,51	1,38E-03	9,92	9,68	4,61E-03	0,54217	0,56627	0,63855	0,6506	8,22	9,27	10,28	11,70	0,94
6508	proteolysis	Biological Process	521	0,45	0,51	2,57E-02	10,71	9,67	2,52E-22	0,33333	0,46341	0,50617	0,5	8,62	10,08	11,28	12,82	0,87
16021	integral to membrane	Cellular Component	493	0,44	0,51	1,36E-02	10,45	9,68	4,68E-16	0,4125	0,4875	0,375	0,5	8,55	9,93	11,04	12,29	0,35
5525	GTP binding	Molecular Function	482	0,56	0,51	9,31E-02	10,73	9,68	5,88E-18	0,38571	0,56338	0,6338	0,66197	8,62	10,00	11,31	12,82	0,93
